# Asymptomatic Spontaneous Pneumopericardium, Pneumomediastinum, and Subcutaneous Emphysema: A Case Report of an Incidental Rare Presentation

**DOI:** 10.7759/cureus.20464

**Published:** 2021-12-16

**Authors:** Mrhaf Alsamman, Sandi Dunn, Shaye Busse, Alan Hamza

**Affiliations:** 1 Internal Medicine, Health Corporation of America-University of Central Florida (HCA-UCF) Consortium, Ocala, USA; 2 Internal Medicine, University of Central Florida College of Medicine, Orlando, USA; 3 Internal Medicine, Ocala Regional Medical Center (ORMC), Ocala, USA

**Keywords:** stroke, emphysema, pneumomediastinum, covid-19, pneumopericardium

## Abstract

Pneumopericardium (PP), pneumomediastinum (PM), epidural pneumatosis, and subcutaneous emphysema (SE) are identified by the existence of free air or gas in the associated spaces. They are normally self-limited unless tension pneumothorax, tension PM, cardiac herniation, air tamponade, and esophageal rupture accompany these disorders. PM and PP can be divided into “spontaneous” or “secondary” based on the preceding etiologies. Spontaneous PM is often extremely rare and benign in course. On the other hand, secondary PM and PP are more common and result from intrathoracic infections, trauma-related esophageal rupture, or tears along the tracheobronchial tree. Our patient presented four days after a fall from a chair and was found to have suffered a stroke, with complete left side paralysis. CT imaging on arrival was significant for PM, PP, and SE, the cause of which remains unclear. The patient was diagnosed with COVID-pneumonia approximately six months prior to presentation. As the COVID-19 pandemic has evolved, several scientific papers have been published reporting infected patients who had developed spontaneous PT, PM, or even PP, in the absence of invasive mechanical ventilation. Is it possible that the spontaneous findings in our patient were COVID-related? Or could the spontaneous PP, PM, and SE be a sequel to the trauma of her fall from a chair? The answer still remains unclear.

## Introduction

Pneumopericardium (PP), pneumomediastinum (PM), epidural pneumatosis, and subcutaneous emphysema (SE) are identified by the existence of free air or gas in the associated spaces. They are normally self-limited unless tension pneumothorax, tension PM, cardiac herniation and air tamponade, and esophageal rupture accompany these disorders [1]. In 1939, Hamman described the first spontaneous PM (SPM). It is seen more in males in the second-fourth decades of life and is normally benign in its course [2]. In 1844, Bricheteau reported the first instance of PP [3]. PM is generally associated with actions that increase intrathoracic pressure. In these cases, it is thought that increased intra-alveolar pressures cause rupture of perivascular alveoli. Then, the air cuts from the ruptured alveoli along the bronchovascular sheaths heading to the mediastinum to produce SPM. This order of events is called the “Macklin effect” [1]. PP is very rare and can be caused by physical trauma to the pericardium, acute, coughing, asthma flare, pregnancy and labor, and fistulas of the pericardium. PP can occur infrequently due to pericarditis, which may be spontaneous, iatrogenic from thoracentesis, pericardiocentesis, or biopsy [1]. Interestingly, our patient did not have any of the aforementioned etiologies. Here we present a case of a 74-year-old female who presented to our facility for left-sided weakness secondary to a stroke and was found to have PP, PM, and SE.

## Case presentation

A 74-year-old female presented to an outside emergency department after a fall from a chair four days earlier and subsequent persistent weakness. Her past medical history is significant for atrial fibrillation not being treated with anticoagulant medications, hypertension, hyperlipidemia, congestive heart failure, type 2 diabetes, and having COVID-19 six months prior, now fully vaccinated. The patient had a CT of the brain which showed no acute findings, and a chest CT that showed PP, PM, and SE of the neck and chest.

She was transferred to our institution for further evaluation, where she was found to be hemodynamically stable. The patient was alert and oriented, had weakness localized to only the left side with 0/5 strength in both the left upper and lower extremity, decreased sensation on the left side, and a left-sided facial droop. A cardiac exam was notable for atrial fibrillation with a rapid ventricular response. Her left lower extremity was erythematous, tender to palpation, had 2+ pitting edema, and had abrasions along the lateral aspect. Of interest, she was also noted to have crepitus along the base of the neck on the right side and at the right chest but did not have any lacerations on the chest or neck. The patient denied any chest pain, palpitations, dyspnea, or cough.

**Figure 1:**
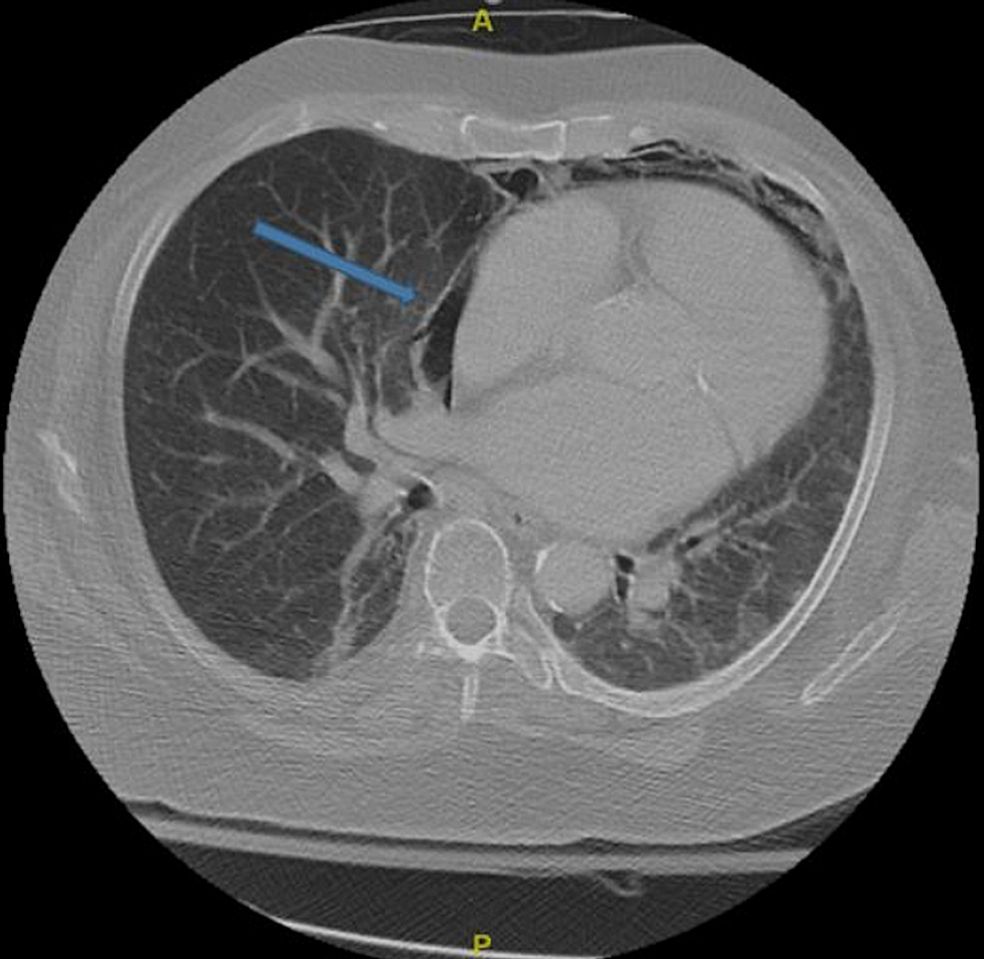
Computed tomography chest showing pneumopericardium (blue arrow)

**Figure 2:**
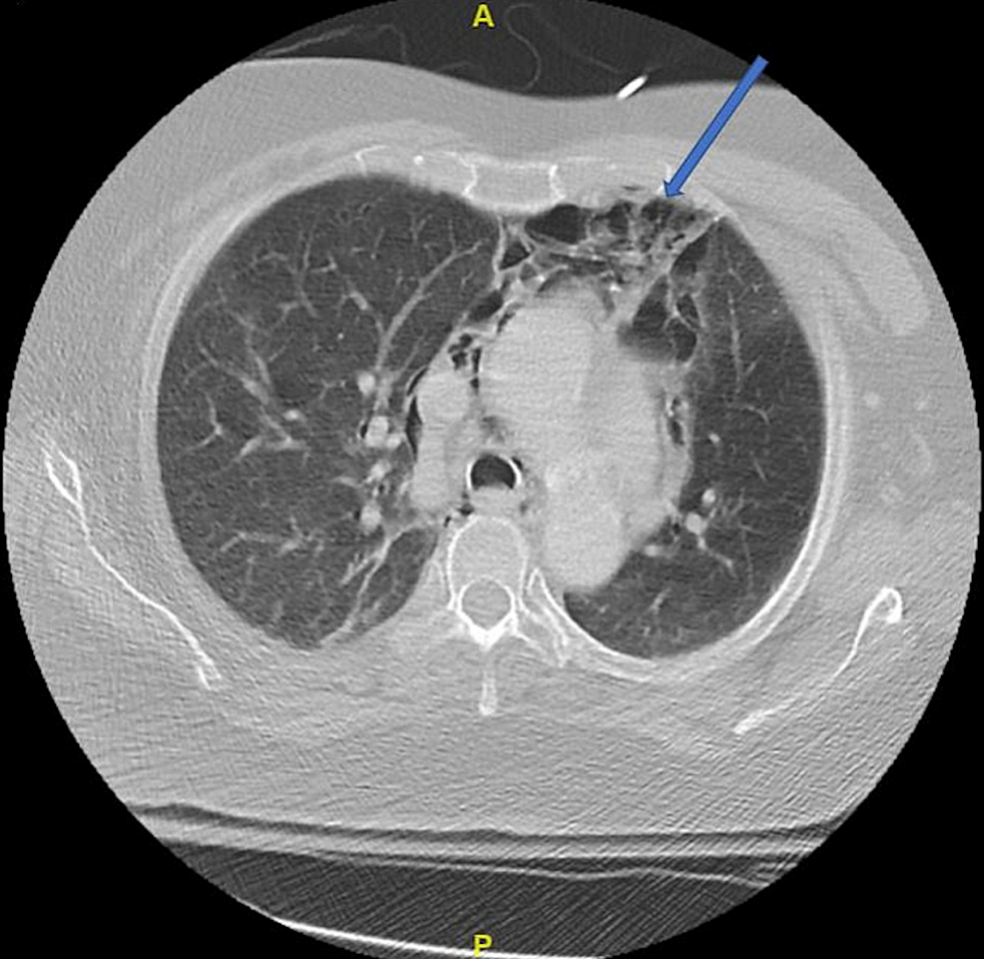
Computed tomography chest showing pneumomediastinum (blue arrow)

**Figure 3:**
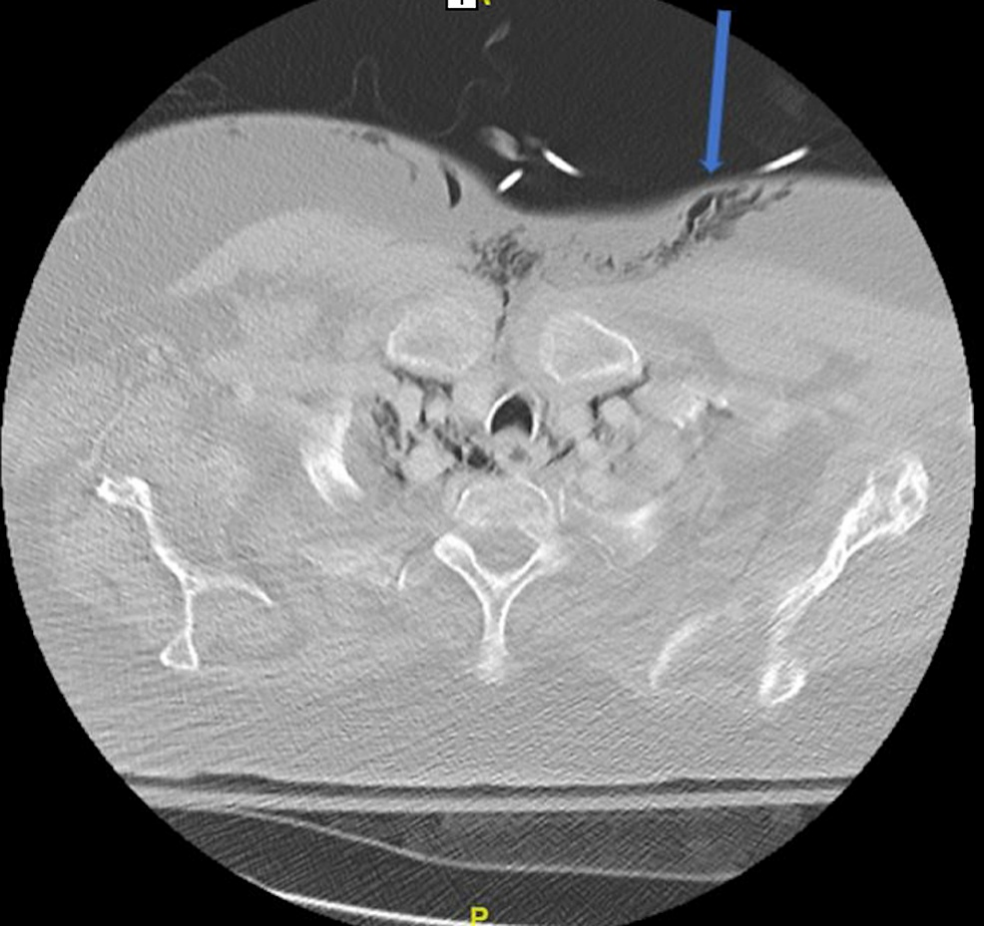
Computed tomography chest showing subcutaneous emphysema (blue arrow)

**Figure 4:**
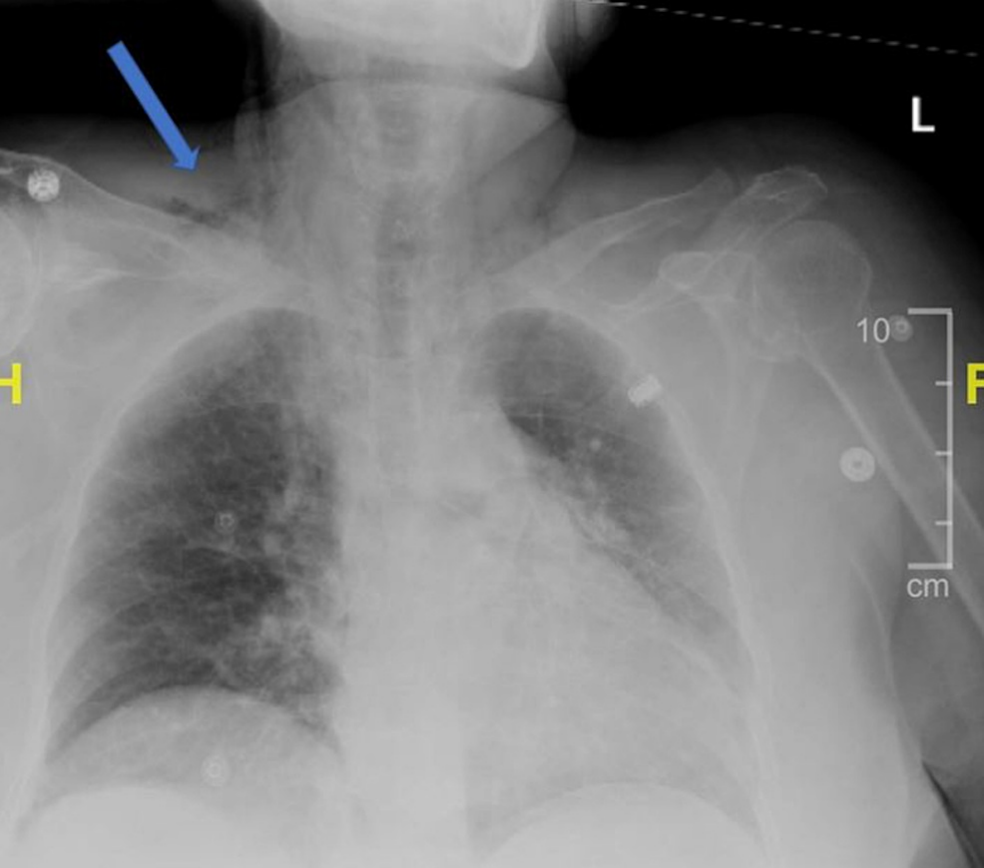
Chest x-ray showing subcutaneous emphysema (blue arrow)

An MRI of the brain confirmed the diagnosis of an early subacute ischemic stroke of the right corona radiata extending to the insular cortex and superior right temporal lobe, and a CTA showed near-total occlusion of the distal right MCA M1 branch. A chest CT showed PP (Figure 1), PM (Figure 2), and SE (Figure 3) unchanged from the CT at the outside ED from the day prior. Chest x-ray revealed PM and SE at the right neck (Figure 4). No draining sinuses or sources for the free air were ever discovered. Additionally, there was no further imaging of the chest done during or after this hospital stay, which would have provided adequate visualization for monitoring of the patient’s asymptomatic PP, PM, and SE.

## Discussion

PP, PM, and SE all refer to the presence of free air in the pericardial cavity, mediastinum, and the subcutaneous tissues of the skin, respectively. PM and PP can be divided into “spontaneous” or “secondary” based on the preceding etiologies. Spontaneous PM is often benign and idiopathic while secondary PM results from intrathoracic infections, traumatic esophageal rupture, or tears along the tracheobronchial tree [4]. Clinical symptoms of PM include dyspnea, odynophagia, pleurisy, retrosternal chest pain, facial and neck pain, dysphagia (which can be secondary to pressurized air from alveolar rupture tracking between tissue planes), or a low-grade fever (possibly secondary to cytokine release) [5,6]. SE is also a common finding and is characterized by the presence of crepitus on palpation along the skin. The Hamman sign, found in 10%-20% of PM cases, is pathognomonic and characterized by systolic crackles at the left sternal border, heard best in the left lateral decubitus position and sometimes reported as the sound of rubbing balloons [2]. Diagnostic modalities including CT chest and chest x-ray, is made by the presence of gas outlining the intrathoracic structures: ring sign (caused by air surrounding the pulmonary artery or either of its main branches), thymic sail (elevation of thymus due to air, mainly seen in the pediatric population), continuous diaphragm sign, double bronchial wall, or air adjacent to hemidiaphragm or spine [7]. Treatment for PM is generally conservative due to typically benign presentation; however, the extent of treatment is dependent on presentation severity. A rare complication from PM is when a large of air is entrapped in the mediastinum, also known as malignant PM, which can cause blockage of the trachea and major vessels present in the mediastinum [8]. This treatment would require decompression with thoracotomy.

PP typically results from trauma, positive pressure through mechanical ventilation, or invasive chest procedures [9]. Diagnostic imaging will reveal air surrounding the cardiac shadow which may possibly cause compression of the heart, rendering it to appear smaller. Important to note, chest radiograph decubitus films will show a shifting of air in PP as the patient changes position in between films, whereas no such shift occurs in PM [3]. The patient with spontaneous PP is usually hemodynamically stable, and it usually heals spontaneously within a few days with conservative management. Further treatment depends on the cause and clinical severity, which is determined by the sum and rate of the accumulation of air in the pericardium [10]. The major complication of PP is pericardial tamponade, which may present with pulsus paradoxus and typical findings of Beck’s triad: hypotension, muffled heart sounds, and distended neck veins secondary to increased jugular venous pressure. Urgent pericardiocentesis or pericardial window is the indicated treatment in such cases.

Our patient presented four days after a fall from a chair and was found to have suffered a stroke, with complete left side paralysis. CT imaging on arrival was significant for PM, PP, and SE, the cause of which remains unclear. Our patient was diagnosed with COVID-pneumonia approximately six months prior to presentation. Of note, as the COVID-19 pandemic has evolved, several scientific papers have been published reporting infected patients who had developed spontaneous PT, PM, or even PP, in the absence of invasive mechanical ventilation [11]. One such scientific paper was a case series, “Spontaneous Pneumomediastinum, Pneumothorax, Pneumopericardium and Subcutaneous Emphysema-Not So Uncommon Complications in Patients with COVID-19 Pulmonary Infection-A Series of Cases” involving 11 COVID-19 positive patients. Another study conducted in Peru, “Spontaneous Pneumopericardium and Pneumomediastinum in Twelve COVID-19 Patients” shed some light on the connection. Both reports, though limited in size, concluded that COVID-19 patients with any of the aforementioned spontaneous phenomena had a poorer outcome. In COVID-19, all mechanisms of lung damage (direct injury, exaggerated inflammatory response and hypercoagulability), could increase the risk of PP, PM, and the possibility of positive pressure injury due to mechanical ventilation [11].

## Conclusions

This case highlights a rare incidental finding of PM, PP, and SE without a definite etiology. Is it possible that the spontaneous findings in our patient were COVID-19 related, especially given the time span since the COVID-19 diagnosis? Or could the spontaneous PP, PM, and SE be a sequel to the trauma of her fall from a chair? The answer still remains unclear. While most spontaneous PP, PM, and SE are self-limited, some complications include pericardial tamponade and obstruction of the trachea due to air entrapment. Therefore, early diagnosis and close monitoring facilitate better outcomes.
